# Development of a Brazilian Food Truck Risk Assessment Instrument

**DOI:** 10.3390/ijerph15122624

**Published:** 2018-11-23

**Authors:** Lígia Isoni Auad, Verônica Cortez Ginani, Eliana dos Santos Leandro, Priscila Farage, Aline Costa Santos Nunes, Renata Puppin Zandonadi

**Affiliations:** 1 Department of Nutrition, Faculty of Health Sciences, University of Brasilia (UnB), Campus Darcy Ribeiro, Asa Norte, Brasilia DF 70910-900, Brazil; vcginani@gmail.com (V.C.G.); elisanleandro@yahoo.com.br (E.d.S.L.); pri_farage@hotmail.com (P.F.); renatapz@yahoo.com.br (R.P.Z.); 2 Department of Pharmacy, Faculty of Health Sciences, University of Brasília (UnB), Campus Darcy Ribeiro, Asa Norte, Brasilia DF 70910-900, Brazil; alinecostasn@gmail.com

**Keywords:** food truck, food safety, street food, checklist, Delphi technique, validation

## Abstract

This study aimed to develop and validate a checklist instrument as a first step for the risk assessment of the hygienic-sanitary practices and conditions of food trucks. We invited sixteen experienced experts in the food safety field to take part in the process. The checklist was designed based on the Codex Alimentarius, Brazilian resolutions Collegiate Board Resolution 216, Brazilian Collegiate Board Resolution 275, Brazilian Federal District Law no. 5.627 and Brazilian Federal District Normative Instruction 11. The preliminary version of the checklist—composed of 29 items (nine sections)—was evaluated by 13 experts. They evaluated the items regarding their importance (content validation) and clarity (semantic evaluation) by the Delphi technique. The criteria for the approval of the content validation (Likert scale from 1 to 5) and semantic evaluation (Likert scale from 0 to 5) processes were as follows: a minimum of 75% agreement among the experts (*W*-values ≥ 0.75) and a mean grade ≥ 4. We performed the complete validation process in three rounds. The final version of the assessment instrument comprised 39 items, following suggestions from experts to add or subdivide some questions. The checklist can be used to conduct inspections of food trucks by health surveillance auditors, of food truck vendors’ decision-making processes and also as a diagnostic tool. The application of this checklist will allow the effective risk assessment of the hygienic-sanitary practices and conditions in food trucks and potentially ensure consumers’ access to safe street food.

## 1. Introduction

According to the World Health Organization [1], street food (SF) is classified as food and beverages prepared and sold on streets and in public places for immediate consumption. The SF sector is booming due to increasing demand for food diversification, availability and accessibility, and the modern lifestyle which is characterized by a lack of time for food preparation and consumption. Every day, about 2.5 billion people worldwide consume street food due to its low cost and convenience [2].

Despite its potential benefits, SF vending activities are mostly in the informal sector and generally escape effective food safety regulation and inspection [3]. Moreover, SF preparation and consumption usually occur in crowded, unhygienic and unsafe places, exposing consumers to potential vehicles for diseases and illnesses of microbial origin [4,5,6,7]. Studies have evidenced several risk factors from SF, including inappropriate storage temperature, ineffective protection from bugs and dust, poor personal hygiene of food handlers and hygienic practices, and a lack of control or inspections [8,9,10,11]. 

SF can be located in schools, car parks, railway stations, office centers, markets, factories and wherever there is a constant stream of customers. There are two main categories of SF: stationary and itinerant vendors [12]. The itinerant SF has been booming with the popularization of food trucks (FTs), boosted by the 2008 recession. In this period, the reduction of purchasing power to eat out and the increase in unemployment among young chefs created a favorable scenario for the rise of FTs [13]. These adapted vehicles for selling food offer a variety of local or international gastronomy with a relatively low initial investment cost and quick financial returns [14].

The FT industry has been increasing worldwide and is now one of the best performing segments of the food service sector. In 2015, the U.S. FT industry was estimated at US$856.7 million, with a growth forecast to US$996.2 million US dollars by 2020 [15]. Similarly, the Brazilian FT industry has been increasing due to the influence of the boom in FTs in the USA. The annual revenue of FTs in Brazil in 2014 was about US$40 billion [16]. The positive performance of FTs is a result of their increasing presence in major cities and as an alternative form of food access with more affordable prices than those offered by restaurants. 

Since FTs represent a particular kind of SF service, they could represent a public health problem. A study performed in California by Faw and Tuttle [17] revealed that 94.73% of the FTs assessed exhibited at least one critical risk factor that contributes to foodborne illness, indicating that mobile FTs demonstrate attributes comparable to fixed food facilities and therefore, would benefit from similar inspection practices. In Brazil, there are no data available in the scientific literature regarding the number of foodborne illnesses related to FTs. It is also important to mention that in the Brazilian Epidemiological Surveillance System (where foodborne diseases are registered) it is not mandatory to register the type of food service nor the place of ingestion of suspect foods [18]. Therefore, there is no registration concerning foodborne illnesses related to Brazilian FTs.

In order to contribute to the safety of FTs, it is important to establish effective strategies to prevent their contamination and to evaluate the food production. Instruments for the verification of non-conformities *in loco* that are related to the occurrence of poor hygiene and handling practices may represent an interesting approach to control the production process and provide safe food, considering the absence of studies that investigate possible strategies to prevent contamination in FTs. The spread of such application systems is due to their ability to improve the food safety of establishments by the awareness of citizens and the accountability of the health sector for ensuring compliance with sanitary regulations [19]. Besides, due to its low cost, high applicability and accessible design, a checklist instrument represents an attractive alternative to conduct inspections of FTs by health surveillance auditors and to the decision-making process of FT vendors willing to comprehend and implement proper food safety practices. It can also be used as a diagnostic tool for the assessment of the hygienic-sanitary practices and conditions in these types of food operations.

The development of an instrument aims to document information in a comprehensive, scientific and straightforward way, allowing identification and diagnosis, so the phenomena of interest must be translated into concepts that can be measured, observed or recorded. The inadequacy of methods for data collection may compromise the validity of the questionnaire conclusions, so it is of great importance to consider some points during this process such as an extensive literature review; the experience of the researcher; the care and monitoring of the formulation of questions/items regarding clarity, consistency, relevance and impartiality; the evaluation of the instrument by experts in the field; and testing to verify the clarity of the instrument and its usefulness for the generation of the desired information [20]. The Delphi technique has been recognized as an efficient method to reach consensus in various fields of study where opinions and judgments of experts and practitioners are necessary [21].

In turn, the validation of the instrument, a key indicator of its quality, may be performed. It is a methodological process related to the precision of the instrument to measure what it purports to measure [22]. Content and semantic validity are steps of the validation process, which can be accomplished by a panel of judges or experts experienced in the subject matter [23]. Content validity is the ability of each item of the instrument to sufficiently represent the content domain while observing grammar, the use of appropriate and correct words and the application of the correct and proper order of words in items [24]. It can provide information on the representativeness and clarity of items and also help improve an instrument through recommendations achieved from the panel of experts [25]. On the other hand, semantic validity is performed in order to identify problems related to the understanding, acceptance and relevance of items, as well as to evaluate the need for adaptations [26].

There are no validated instruments available for assessing the hygienic-sanitary conditions of FTs and, therefore, there is a need for diagnostic instruments to evaluate these vehicles. Thus, the aim of the current research was to perform content validation and semantic evaluation of a checklist instrument for the assessment of food safety conditions and practices in FTs.

## 2. Materials and Methods

### 2.1. Development of the Instrument to Assess the Hygienic and Sanitary Practices and Conditions of Food Trucks (Qualitative Analysis)

**Figure 1:**
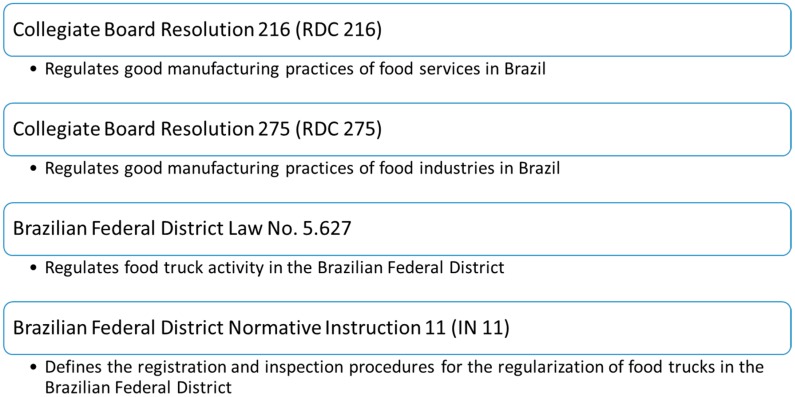
Resources used to develop the checklist.

The checklist instrument was designed after an extensive literature review and consideration by the researchers based on their experience. The technical and legal resources used to elaborate the preliminary version of the checklist are listed in Figure 1. 

This checklist was developed based on current food safety regulations, focusing on the most significant factors to be controlled to prevent foodborne diseases. Topics and items from the Brazilian legislation RDC 216 [27] and RDC 275 [28], which are based on the General Principles of Food Hygiene of the Codex Alimentarius [29], were carefully evaluated and those relevant to the food safety of food trucks were selected and adapted for the initial version of the checklist, despite the fact that these documents do not specifically address the FT environment. However, the food safety and quality of FTs are based on a prerequisites program implemented by the vehicle that satisfies the minimum of good manufacturing practices, as proposed by the local Brazilian legislation No. 5.627 [30]. Significant topics of IN 11 [31] were also selected for the checklist and adapted to account for the reality of FTs in Brazil.

The preliminary version of the instrument was composed of 29 items, divided into nine sections. The sections included vehicle structure and adjacent areas, equipment and kitchenware, hygiene and cleanliness, food and water storage, food and water preparation and handling, residue handling, food handlers, pest and vector control, and documentation (Good Manufacturing Practices Manual and Standard Operation Procedures).

The checklist is comprised of open-ended questions with identification data to characterize the FT (name, address, owner, among others). The nine sections of the instrument are designed with closed-ended questions that require a “Yes/No/Not Applicable” answer. 

### 2.2. Validation Process (Objective Evaluation)

The validation process, characterized as the objective evaluation, was performed using the Delphi technique with some adaptations. Widely employed and accepted in various fields to achieve convergence of opinion of experts in certain topic areas, the Delphi method is designed as a group communication process which allows each participant an opportunity to conduct detailed examinations and discussions of a specific issue by using a series of questionnaires, aiming to generate a consensus of the respondent group [32].

A total of 16 Brazilian experts in various areas relevant to this topic (health surveillance, food safety, street food, food microbiology, risk analysis, quality control) were invited by e-mail to participate. Experts were PhD and/or post-graduate professors and health surveillance authorities with extensive experience in the research field. A total of 13 experts were available for the study. The experts received the necessary information and guidance on the checklist method of evaluation.

The Survey Monkey^®^ platform was used to apply the checklist document, as well as to provide the experts’ feedback in regard to the evaluation performed and the final results of the content and semantic validation analysis.

### 2.3. Content Validation and Semantic Evaluation

The Survey Monkey^®^ platform was used to conduct the validation process, which consisted of content validation and semantic evaluation simultaneously. Evaluation criteria for the checklist items were presented on the first page of the questionnaire by a covering letter, indicating the beginning of the first round.

Experts were asked to evaluate each item considering its importance for the assessment of food safety in food trucks using a five-point Likert scale, as follows: (1) “I totally disagree with the item”; (2) “I partially disagree with the item”; (3) I neither agree nor disagree with the item”; (4) “I partially agree with the item”; and (5) “I totally agree with the item”.

For the semantic evaluation, experts were asked to evaluate each item regarding its clarity, considering their level of understanding of the item. A six-point Likert scale was used for this step, as follows: (0) “I did not understand it at all”; (1) “I understood it a little”; (2) “I somewhat understood it”; (3) “I understood almost everything, but I had some questions”; (4) “I understood almost everything”; and (5) “I understood it perfectly”. 

Answers from 0 to 3 indicate insufficient understanding, requiring the item to be rewritten [33]. Experts were asked to suggest changes in cases of poor understanding or unsuitable language of an item. These opinions were used to reformulate the item for further evaluation. 

Two stages of evaluation were performed for both content validation and semantic evaluation. In the first round, 13 of the 16 experts agreed to participate, while nine respondents participated in the last stage.

### 2.4. Data Analysis

The evaluation of the importance (content validation) and clarity (semantic evaluation) of each item was achieved by the mean grade and the degree of agreement among experts. The mean grade was calculated as the average grade of the answers. Items had to achieve a mean ≥ 4 for importance and clarity evaluations to be maintained in the instrument.

The degree of agreement among experts was obtained through Kendall’s (*W*) coefficient of concordance, which ranges from 0 to 1. High *W*-values (*W* > 0.66) indicate agreement among the experts and low *W*-values suggest different standards of evaluation applied by the experts [20]. Therefore, a minimum of 75% agreement among experts (*W*-values ≥ 0.75) for both content validation and semantic evaluation was required for the approval of each item.

Items that did not meet these requirements were considered unimportant for the assessment of the food safety of food trucks and, for that reason, were excluded from the instrument. Items considered unclear were rewritten and then subject to further evaluation by the experts. Suggestions and observations collected from the panel of experts were also considered and incorporated into the final version of the instrument. 

## 3. Results

**Figure 2:**
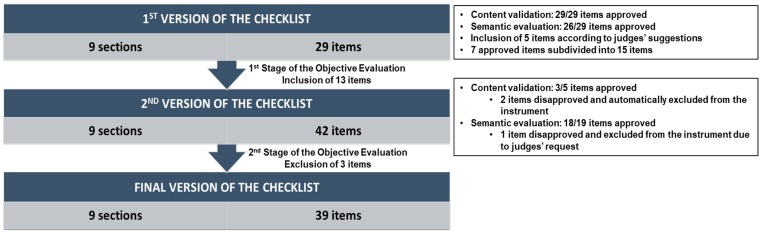
Stages of the content validation and semantic evaluation process.

The validation process is summarized in Figure 2.

### 3.1. First Round: Content Validation and Semantic Evaluation

All 29 items (100%) of the first version of the instrument achieved a mean grade ≥ 4 and the minimum of 75% agreement among experts (*W*-values ≥ 0.75) in the content validation and were, therefore, approved.

As for the semantic evaluation, a total of 26 items (89.6%) met the requirements needed for approval. Two of the three remaining items not approved were from the “Vehicle Structure and Adjacent Areas” section and the other one was from the “Hygiene and Cleanliness” section. Item 1.3 (regarding the design of the vehicle structure) achieved a mean grade of 3.64 and a *W*-value of 54.5%. Items 1.7 (regarding the cleanliness of the adjacent area of the food truck) and 3.1 (regarding the use of chemical sanitizers to clean the vehicle structure and the equipment and kitchenware) both achieved a mean grade of 4.0 but a Kendall’s coefficient concordance (Kendall’s *W*) of 63.6%. Thus, these three items were considered insufficiently understandable by the experts and it was necessary to adjust their wording.

The mean grades and *W*-values of each section, considering the means of all items, for the content validation and semantic evaluation are presented in Table 1.

Although 26 items were approved in the content validation and semantic evaluation in the first stage, it was suggested by the experts that some of them should be rewritten to add relevant information and/or to make the writing less ambiguous. They also stated that some other items required the assessment of many requisites, therefore suggesting their subdivision into more than one item. 

Hence, considering the judges’ observations, five new items were included in the “Food and Water Preparation and Handling” section, and seven items previously approved were subdivided into 15 items. In the “Vehicle Structure and Adjacent Areas” section, items 1.1 (regarding the surface material of the vehicle structure and their condition) and 1.3 (regarding the design of the vehicle structure) were subdivided into items 1.1 and 1.2, and items 1.3 and 1.4, respectively, while in section 2 (Equipment and Kitchenware), item 2.3 (regarding the use of a thermometer to check food temperature) was subdivided into items 2.3 and 2.4. Item 3.1 (regarding the use of chemical sanitizers to clean the vehicle structure and the equipment and kitchenware) was subdivided into items 3.1 and 3.2 in the “Hygiene and Cleanliness” section. In the “Food and Water Storage” section, item 4.1 (regarding food storage condition and identification) was subdivided into items 4.1 and 4.2. Finally, in section 7 (Food Handlers) item 7.1 (regarding health and hygiene of food handlers) was subdivided into items 7.1, 7.2 and 7.3. 

The new items (5.3, 5.4, 5.5, 5.6 and 5.7) in the “Food and Water Preparation and Handling” section were submitted to content validation and semantic evaluation, while the subdivided items (1.1, 1.2, 1.3, 1.4, 2.3, 2.4, 3.1, 3.2, 4.1, 4.2, 7.1, 7.2, 7.3) and item 1.7 were resubmitted only to semantic evaluation, since their importance was already attested in the content validation process.

Thus, a total of 19 items were submitted to a new semantic evaluation, of which five were also submitted to a new content validation in stage 2.

### 3.2. Second Round: Content Validation and Semantic Evaluation

The newly included items 5.3, 5.4, 5.5, 5.6 and 5.7 were submitted to both content validation and semantic evaluation. Two of those items (5.3 and 5.4) were automatically removed from the checklist (mean grade < 4 in content validation), while three (60%) were maintained (mean grade ≥ 4 and *W*-value ≥ 75% in both content validation and semantic evaluation). One of the approved items (5.5) was incorporated into item 4.3, following the experts’ suggestion.

From the other 14 items resubmitted to semantic evaluation, only item 2.3 was not considered sufficiently understandable. Although previously approved in content validation, this item was excluded from the instrument following the request of the experts. All other items reformulated considering the judges’ comments in stage 1 were approved in this new version and, therefore, retained in the instrument.

With the approval in content validation and semantic evaluation of the recently added items and the new versions of the other items approved in the semantic evaluation according to the judges’ additional comments, the final version of the instrument was successfully completed, with a total of 39 items divided into nine sections. The final instrument should take about 15–20 minutes to complete.

It is noteworthy that the process of objective evaluation was fully performed in Brazilian Portuguese, which is the instrument’s original language version. The complete checklist, found in Supplementary File 1, was translated into English to facilitate readers’ understanding.

## 4. Discussion

In this study, the opinion of an expert group using the Delphi technique was chosen to develop a scientifically founded instrument to assess food safety of FTs, primarily due to the lack of an appropriate or available validated and reliable instrument for this purpose. The objective evaluation performed in this study is a key element in developing a reliable and valid instrument, since it ensures the importance and proper understanding of the items which, in turn, will directly impact food safety assessments of FTs. The final version of the instrument was carefully revised and included all items considered important and comprehensive by the experts.

This study has some potential limitations, namely the sample size and response rate of the expert panel. In our study, 13 experts participated in the first round, with a response rate of 81%, while nine participated in the second round, with a response rate of 69%. Despite repeated attempts, the number of experts could not be increased, which can be explained by some potential participants lacking practical experience with food trucks and the pioneering nature of this study. Consequently, due to the limited number of qualified participants, we were unable to sustain a high response rate in all iterations. While the number of experts is not large, there is no agreement on the panel size for Delphi studies, since the sample is purposefully selected and also depends on the scope of the problem [34]. Similarly, although the response rate is not high, we were able to maintain a response rate of 70% in all iterations to sustain the rigor of the Delphi technique, as recommended by Hasson et al. [35]. 

Despite these limitations, the Delphi technique provided consistent results and offered several advantages in this study. The Delphi tool, along with the Survey Monkey^®^ platform, provided anonymity and confidentiality to the expert panelists group, facilitating group communication and sharing of information among individuals from geographically diverse locations in a cost-effective manner [34]. Also, due to its iterative nature, the Delphi process provided a substantial amount of time to each participant to freely and impersonally express their ideas and opinions [36]. Similarly, in a study performed by Ceniccola et al. [37], the Delphi technique and Survey Monkey^®^ platform were used to guide the stages of expert evaluation, enabling an organized and efficient interaction among experts. 

The criteria used to qualify an individual to compose a Delphi panel may vary due to the context, scope and aims of the particular study [38]. The selection of the expert panel was another positive aspect of our study. Besides considering their experience (“expertise”) and knowledge (“knowledgeability”), experts should satisfy some requirements, such as having sufficient time and willingness to participate in the process and having effective communication skills. Therefore, the criteria used to identify potential experts included authors of publications in high impact factor journals and with considerable experience in the research field of this study. The selection of experts with a diverse vocational education and background (microbiologists, nutritionists, biologists), and thus with different perspectives, aimed to form a broad Delphi panel. Farage et al. [39] also considered experts’ experience and willingness to collaborate when selecting the panel for the development and validation of an instrument for the control of gluten contamination in food services in Brazil.

Another issue the Delphi technique successfully addressed in our study was the level of consensus. Agreement was reached by the calculation of mean grades and Kendall’s coefficient of concordance (Kendall’s *W*) for the process of both content validation and semantic evaluation. Kendall’s coefficient of concordance ranges from 0 to 1 and indicates the level of consensus reached by the panel (strong consensus *W* > 0.7; moderate consensus = 0.3–0.7; and weak consensus *W* < 0.3) [40]. For the current study, an agreement was determined by a mean grade ≥ 4, calculated based on the experts’ opinion, and a minimum of 75% agreement among experts (*W* > 0.75), indicating strong consensus reached by the panel. Strong agreement of the expert panel was expected, since the general principles and requirements concerning food safety are well established and should be applied in food production, regardless of the environment. 

Thus, the final instrument contemplates universal core issues within food safety, based on the Five Keys to Safer Food Manual [41] and the General Principles of Food Hygiene of the Codex Alimentarius [29]. The first and second sections (Vehicle structure and Adjacent Areas and Equipment and Kitchenware, respectively) include items regarding the location, design, layout and construction of the vehicle, as well as its fixtures, fittings and equipment, which are vital to ensure that the eating establishment can operate under hygienic conditions and produce food safely. Poorly designed and constructed facilities and equipment are potential sources of physical, chemical and microbiological hazards, which can cause illness or injury to consumers and so must be prevented or minimized [42,43]. The structure and equipment should also be designed to facilitate maintenance and good sanitation practices, as well as to minimize potential harborages of pests, which are evaluated in sections three (Hygiene and Cleanliness) and eight (Pest and Vector Control), respectively.

Sections four and five are composed of items concerning food and water storage and handling. Food unproperly stored can cause food spoilage or contamination and possibly food poisoning for the consumer. According to a World Health Organization study [44], inappropriate temperatures during the food process were responsible for 45.6% of reported cases of foodborne diseases, of which poor refrigeration accounted for 23.5% of cases and inappropriate storage temperatures of leftover or recently cooked meals accounted for 12.6% of cases.

The surrounding environment may pose a risk to safe food hygiene and, therefore, items regarding residue handling are assessed in section six. Section seven consists of items regarding food handlers, which are another potential source of food contamination since they work in unsanitary conditions with little or no infrastructure support. Besides, satisfactory knowledge of food safety does not necessarily translate into strict hygienic practices during the processing and handling of food products [45]. The last section of the checklist deals with items concerning documentation.

This study not only reinforces the importance of the use of rigorous methods for expert consensus on the development of validated instruments, but also highlights the urgent need for an assessment tool for the evaluation of food safety conditions and practices in food trucks. Due to its low cost, high applicability and accessible design, the proposed instrument represents an attractive alternative to conduct inspections in food trucks by health surveillance auditors and to the decision-making process of food truck vendors willing to comprehend and implement proper food safety practices. It can also be used as a diagnostic tool for the assessment of the hygienic-sanitary practices and conditions in these types of food operations. 

This research is part of a larger study, which is currently in progress. The checklist will be applied in food trucks of the Federal District, along with the collection of food samples for microbiological examination. Data obtained will be statistically analyzed to determine which items/sections pose a high risk to producing safe food and their relation to the level of potential food safety risk. The application of this instrument will also allow the implementation of a food safety grading system, in which food trucks will receive a score based on their sanitary quality or the degree of compliance with food safety regulations.

## 5. Conclusions

A food safety assessment instrument for food trucks was designed and validated, considering content and semantics, by the Delphi method with some adaptations. A published description of the methods and the results of the development and validation processes of this instrument will promote scientific discussion and may assist the implementation of similar scientifically founded systems in the food safety field. Further studies are necessary to assess the psychometric properties of the instrument, such as reliability, a main quality criterion which consists of the ability to reproduce a consistent result from different observers presenting aspects on coherence, stability, equivalence and homogeneity. The application of the instrument in food trucks is also important to adequately assess food trucks with regard to their hygienic-sanitary practices and conditions. Such actions are fundamental to ensure consumer access to safe street food and ultimately to protect public health.

## Figures and Tables

**Table 1:** Experts’ evaluation of the checklist with mean grades and Kendall’s coefficient of concordance (*W*) of the checklist sections in the first round of objective evaluation.

Section of the Checklist	Content Validation (Mean Grade ± SD *)	Content Validation (*W*-Value)	Semantic Evaluation (Mean Grade ± SD *)	Semantic Evaluation (*W*-Value)
Vehicle Structure and Adjacent Areas	4.74 ± 0.30	0.96	4.76 ± 0.15	0.92
Equipment and Kitchenware	4.79 ± 0.25	0.97	4.83 ± 0.17	0.96
Hygiene and Cleanliness	4.81 ± 0.20	0.98	4.79 ± 0.20	0.93
Food and Water Storage	4.79 ± 0.21	0.96	4.87 ± 0.17	0.98
Food and Water preparation and Handling	4.86 ± 0.14	0.94	5.00 ± 0.00	1.00
Residue Handling	4.82 ± 0.27	0.96	4.75 ± 0.32	0.96
Food Handlers	4.86 ± 0.00	1.00	5.00 ± 0.00	1.00
Pest and Vector Control	4.57 ± 0.26	0.89	5.00 ± 0.00	1.00
Documentation	4.78 ± 0.23	0.92	4.71 ± 0.29	0.94

* Standard Deviation.

## Supplementary Materials

The following are available online at http://www.mdpi.com/1660-4601/15/12/2624/s1, File 1: Checklist for the Food Safety Assessment of Food Trucks.
