# Supplementary material for: Development of a Brazilian Food Truck Risk Assessment Instrument
Source: Int J Environ Res Public Health. 2018 Nov 23;15(12):2624. doi: 10.3390/ijerph15122624 (PMC6313429; doi:10.3390/ijerph15122624)
Supplement: Supplementary file 1 [file ijerph-15-02624-s001.pdf]

Checklist for the Hygienic-Sanitary Diagnostic of Food Trucks

Legend

Y - Yes N - No NA - Not Applicable OBS - Observation

**Number:**	**Year:**
**Food Truck:**	
**Owner:**	
**Email/Phone:**	

**1.**	**Vehicle Structure and Adjacent Areas**	Y	N	NA	OBS
1.1	Internal surfaces (walls, floor and ceiling) are of smooth, resistant, impermeable material that allows easy sanitation. They are properly conserved, free of cracks, drips, molds and dehulling.				
1.2	Internal surfaces (walls, floor and ceiling) are designed to facilitate maintenance, cleaning and, when appropriate, disinfection operations (i.e., no hard-to-reach quarters and areas, without equipment located in order to prevent or hinder cleaning).				
1.3	Surfaces in direct contact with food are smooth, resistant, waterproof and easily sanitized (stainless steel, mineral solid surface and similar). They are properly conserved, free of dirt, cracking and peeling.				
1.4	Surfaces in direct contact with food are designed to facilitate maintenance, cleaning and disinfection operations, with no hard-to-reach quarters and areas, with no equipment located to prevent or hinder cleaning.				
1.5	Fixtures are protected against explosion and accidental drops. They are properly conserved (without cracks, rust and exposed wiring) and allow sufficient lighting to carry out the activities.				
1.6	Electrical installations are embedded or protected in external and intact pipes to allow proper sanitation.				
1.7	The water tank is of smooth, waterproof and resistant material (polyethylene, polypropylene, stainless steel and similar), cleaned, rust free, suitable for carrying out activities (hygiene of hands, utensils, equipment and surfaces), kept capped and stocked with drinking water.				
1.8	The area occupied by the food truck and the eating area (with removable furniture, such as tables and chairs) are kept free of dirt, food residues or other types of waste, as well as the presence of pests and urban vectors during the activity and immediately after its closure.				
**2.**	**Equipment and Kitchenware**	Y	N	NA	OBS
2.1	Equipment and utensils are properly conserved and clean. They allow proper cleaning, are of non-toxic material and, if necessary, are heat resistant and with a protection and safety device.				

2.2 Equipment and utensils used at all stages of food preparation are stored and are washed after use and sanitized before use or are disposable. They are protected from dust and contaminants when stored.				
2.3 Equipment (refrigerator, freezer, plate, fryer and similar) has an external thermometer. If not, there is a calibrated food thermometer that records temperature.				
**3. Hygiene and Cleanliness**	Y	N	NA	OBS
3.1 Internal surfaces (walls, floors and ceilings) are cleaned using materials and products (sanitizing agents, disinfectants and similar) specific for this purpose, with procedures according to Work Instruction (WI), Standard Operating Procedures (SOP) or the manufacturer's instructions.				
3.2 Surfaces used in food preparation or in direct contact with food are cleaned using materials and products (sanitizing agents, disinfectants and similar) specific for this purpose, with procedures according to Work Instruction (WI), Standard Operating Procedures (SOP) or the manufacturer's instructions, before and after each use.				
**4. Food and Water Storage**	Y	N	NA	OBS
4.1 Ingredients, raw material and pre-prepared and ready-to-eat foods are stored off the floor, in a clean space and separately, away from disposable and cleaning materials.				
4.2 Ingredients, raw material and pre-prepared and ready-to-eat foods are correctly labeled with an expiration date in clean and undamaged packaging.				
4.3 Perishable ingredients, raw material and pre-prepared and ready-to-eat foods are stored separately according to food groups (milk and dairy products, meat, vegetables) and categories (raw and cooked, sanitized and unsanitized), under refrigeration or freezing temperature.				
4.4 Semi-perishable ingredients, raw material and pre-prepared and ready-to-eat foods are stored and separated according to food groups (cereals, legumes, sugars, oils) and preserved as instructed by the manufacturer or supplier.				
**5. Food and Water Preparation and Handling**	Y	N	NA	OBS
5.1 There are no crosses, i.e., there is no direct or indirect contact between raw and cooked food or between sanitized and unsanitized food at all stages of food preparation (pre-preparation, preparation, distribution).				
5.2 Ingredients, raw material and pre-prepared and ready-to-eat foods that have been fractionated or transferred from their original packaging are packed in containers with a lid, waterproof, washable and non-toxic material or plastic bags suitable for food, sealed and of first-use, and				

	identified by name, date of preparation / fractionation / manipulation and expiration date.				
5.3	Perishable ingredients, raw material and pre-prepared and ready-to-eat foods are exposed to room temperature only for the minimum time required for handling, with maximum preparation time at room temperature of 30 minutes.				
5.4	The thawing of ingredients, raw material and pre-prepared and ready-to-eat foods is carried out in refrigerated equipment or in a microwave oven when the food is immediately subjected to cooking.				
5.5	Ingredients, raw material and food submitted to cooking reach temperature and time of 65ºC/15min, 70ºC/2min, 74ºC in the geometric center or other combinations of the binomial time / temperature that ensure hygienic-sanitary quality.				
5.6	Fruits, vegetables and legumes, if not previously sanitized, are submitted to sanitation, using products regularized by ANVISA or Ministry of Health, with POP or IT established and respected in at least three stages (washing, sanitization and rinsing).				
5.7	Oils and fats used for frying have their quality guaranteed by means of temperature control (heated to a maximum of 180ºC), observation of sensorial characteristics (no change in color, odor and taste and no presence of smoke and foam) and/or use of a pH indicator strip.				
5.8	In the case of re-use of oils and fats, they must be previously filtered (in proper filters or white cloth boiled for 15 minutes), packed in identified and capped containers and, when the interval between uses is long, stored under refrigeration.				
**6.**	**Residue Handling**	Y	N	NA	OBS
6.1	Waste collectors in the internal area of the vehicle are clean and kept covered with bags suitable for the activity. They are hands-free lids (with pedal, sensor or similar) and are emptied before reaching their maximum capacity and bags are discarded in an appropriate place.				
6.2	Waste collectors in the external area of the vehicle are clean and kept covered with bags suitable for the activity. They are lid hands-free (with pedal, sensor or similar) and are emptied before reaching their maximum capacity and discarded bags in an appropriate place.				
6.3	Waste water is kept in a container exclusively for this purpose, kept closed and away from food, and discarded in a place indicated by the local water and sewage company.				
6.4	Residual oils and fats are packed in rigid containers and capped until discarded in accordance with applicable environmental legislation.				

**7.**	**Food Handlers**	Y	N	NA	OBS
7.1	The personal hygiene routine of handlers includes the use of complete uniforms (footwear, pants, lab coats or shirts, without any apparent personal clothing), preserved, cleaned and used exclusively during food handling operations.				
7.2	The personal hygiene routine of manipulators includes the use of hair trapped and protected with a cap. In the case of a beard and mustache, a mask should be used. Clean nails, short, no enamel or base. Without the use of adornments (necklaces, amulets, bracelets, ribbons, earrings, nails and false eyelashes, piercing on exposed areas, watches, rings and rings) during food handling operations.				
7.3	Handlers with cutaneous lesions and wounds or symptoms of diseases/infections (e.g., respiratory, gastrointestinal, ocular) are excluded from food handling operations.				
7.4	Handlers do not smoke, sing, whistle, sneeze, spit, cough, eat or practice other acts that may contaminate food during food handling operations.				
7.5	Handlers do not manipulate money while handling food.				
7.6	Handlers carefully wash their hands when they arrive at work, before and after handling food, after any interruption of service, after touching contaminated materials, after using the toilets and whenever necessary.				
7.7	Handlers use disposable gloves in place of utensils to handle only ready-to-eat foods and previously sanitized fruits and vegetables, replacing them and disposing of them as soon as they discontinue the procedure and before touching another food or surface that is not part of the preparation.				
**8.**	**Pest and Vector Control**	Y	N	NA	OBS
8.1	There is a set of effective and continuous control actions to prevent the attraction, shelter, access and proliferation of vectors and pests in the internal area of the vehicle.				
**9.**	**Documentation**	Y	N	NA	OBS
9.1	Existence of a food handler proven to be able to implement and supervise Good Practices.				
9.2	Existence of a Manual of Good Practices and Standard Operating Procedures (SOP) of the whole process (reception of raw materials, storage, pre-preparation, preparation and distribution, hygiene and health, hygiene of facilities, equipment and utensils; water reservoir, waste control, vector control and pest control).				
